# Postoperative fever predicts poor prognosis of gastric cancer

**DOI:** 10.18632/oncotarget.15979

**Published:** 2017-03-07

**Authors:** Fan Feng, Yangzi Tian, Xuewen Yang, Li Sun, Liu Hong, Jianjun Yang, Man Guo, Xiao Lian, Daiming Fan, Hongwei Zhang

**Affiliations:** ^1^ Division of Digestive Surgery, Xijing Hospital of Digestive Disease, Fourth Military Medical University, Xi’an, Shaanxi, China; ^2^ Department of Dermatology, Xijing Hospital, Fourth Military Medical University, Xi’an, Shaanxi, China

**Keywords:** gastric cancer, postoperative fever, prognosis

## Abstract

Data about prognostic value of postoperative fever in gastric cancer was lacking. Thus, the present study aims to investigate the prognostic value of postoperative fever in gastric cancer. From September 2008 to March 2015, 2938 gastric cancer patients were enrolled in the present study. Clinicopathological features were recoded. The association between postoperative fever and prognosis of gastric cancer were analyzed. There were 2294 male (78.1%) and 644 female (21.9%). Seven hundred and fifty-six patients suffered from fever. Among them, the duration of fever less than 48h occurred in 508 cases, and duration of fever over 48h occurred in 248 cases. Univariate and multivariate analysis showed that postoperative fever was an independent risk factor for prognosis of gastric cancer (*P* < 0.001). For the entire cohort, duration of fever over 48h was significantly associated with decreased survival (*P* < 0.001). In subgroup analysis, duration of fever over 48h was significantly associated with poor prognosis of stage I and II gastric cancer (both *P* < 0.001). However, postoperative fever was not associated with the prognosis of stage III gastric cancer (*P* = 0.334). Considering the type of gastrectomy, postoperative fever was not associated with the prognosis of patients with proximal (*P* = 0.318) and distal gastrectomy (*P* = 0.806), but duration of fever over 48h was significantly associated with poor prognosis of patients with total gastrectomy (*P* = 0.004). In conclusion, postoperative fever was associated with poor prognosis of gastric cancer.

## INTRODUCTION

Gastric cancer is the fifth commonest malignancies and the third leading cause of tumor related death in the world [1]. Moreover, it is the second most common cancer in China [2]. Radical gastrectomy remains the optimal treatment for non-metastatic gastric cancer. Although with the rapid improvement in surgical techniques and adjuvant therapy, the prognosis of advanced gastric cancer is still discouraging [3].

Fever is a common event after major abdominal surgery [4], with an incidence ranges from 13 to 39 percent. It is well known that postoperative fever could result in anxiety of patients, prolonged hospitalization and increased hospital costs. However, data about the prognostic value of postoperative fever in cancer patients was limited. Up to now, only a few studies investigated the prognostic value of postoperative fever in breast cancer [5, 6] and colorectal cancer [7-9]. However, the findings were controversial. Moreover, the prognostic value of postoperative fever in gastric cancer patients has not been reported up to now.

Given this situation, the present study aims to investigate the prognostic value of postoperative fever in gastric cancer patients after radical gastrectomy.

## RESULTS

The clinicopathological characteristics were shown in Table 1. There were 2294 male (78.1%) and 644 female (21.9%). The patient age ranged from 20 to 90 years (median, 58 years; mean, 57.4 years). The follow up time ranged from 1 to 75 months (median, 21 months; mean, 26.8 months). Seven hundred and fifty-six patients suffered from postoperative fever. Among them, the duration of fever less than 48 h occurred in 508 cases, and duration of fever over 48h occurred in 248 cases. The 1-, 3- and 5-year overall survival rate for the entire cohort was 90.0%, 67.4% and 59.7%, respectively. The 1-, 3- and 5-year overall survival rate for patients with fever less than 48h was 88.2%, 66.0% and 57.2%, respectively. The 1-, 3- and 5-year overall survival rate for patients with fever over 48h was 81.2%, 58.2% and 44.8%, respectively. Postoperative fever was associated with gender, tumor location, type of resection, tumor depth, lymph node metastasis, and tumor stage.

**Table 1:** Comparison of clinicopathological characteristics

Characteristics	No fever (n=2182)	Fever ≤48h (n=508)	Fever >48h (n=248)	*P* value
Gender				0.011
Male	1681(73.3)	422 (18.4)	191 (8.3)	
Female	501(77.8)	86 (13.4)	57 (8.9)	
Age				0.917
≤60	1297(74.2)	305 (17.5)	145 (8.3)	
>60	885(74.3)	203 (17.0)	103 (8.6)	
Tumor location				<0.001
Upper third	609 (69.0)	189 (21.4)	85 (9.6)	
Middle third	369 (75.2)	71 (14.5)	51 (10.4)	
Lower third	1037 (78.1)	208 (15.7)	83 (6.3)	
Cross or entire	167 (70.8)	40(16.9)	29 (12.3)	
Tumor size (cm)				0.231
≤5	1548(74.9)	356 (17.2)	163 (7.9)	
>5	634(72.8)	152 (17.5)	85.(9.8)	
Type of resection				<0.001
Proximal	166 (65.6)	63 (24.9)	24 (9.5)	
Distal	984 (77.9)	200 (15.8)	79 (6.3)	
Total	1032 (72.6)	245 (17.2)	145 (10.2)	
Pathological type				0.412
Well differentiated	231 (74.8)	51 (16.5)	27 (8.7)	
Moderately differentiated	551 (72.3)	139 (18.2)	72 (9.4)	
Poorly differentiated	1295 (75.6)	286 (16.7)	133 (7.8)	
Signet ring cell or Mucinous	105 (68.6)	32 (20.9)	16 (10.5)	
Tumor depth				<0.001
T1	432(78.3)	81 (14.7)	39 (7.1)	
T2	337(71.9)	81 (17.3)	51 (10.9)	
T3	736(69.2)	217 (20.4)	110 (10.3)	
T4	677(79.3)	129 (15.1)	48 (5.6)	
Lymph node metastasis				0.005
N0	796(75.3)	177 (16.7)	84 (7.9)	
N1	376(67.6)	117 (21.0)	63 (11.3)	
N2	380(76.8)	84 (17.0)	31 (6.3)	
N3	630(75.9)	130 (15.7)	70 (8.4)	
Tumor stage				0.013
I	561(76.0)	119 (16.1)	58 (7.9)	
II	597(70.0)	166 (19.5)	90 (10.6)	
III	1024(76.0)	223 (16.6)	100 (7.4)	

**Figure 1:**
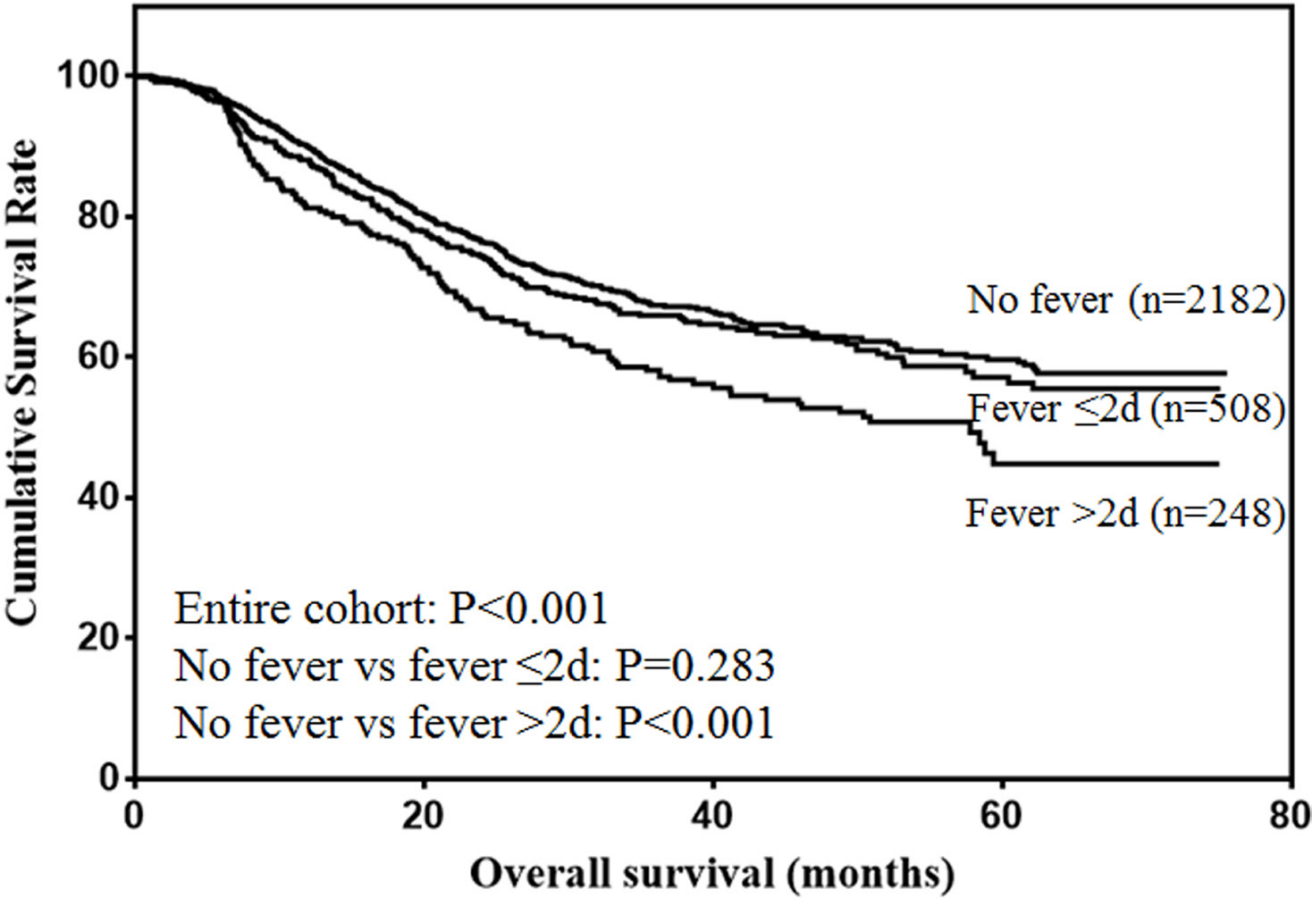
Overall survival of gastric cancer patients

The risk factors for the prognosis of entire cohort analyzed by univariate analysis were shown in Table 2. The results showed that age, tumor size, type of resection, pathological type, tumor depth, lymph node metastasis, tumor stage and fever were risk factors for the prognosis of gastric cancer. Multivariate analysis showed that age, tumor size, tumor depth, lymph node metastasis and fever were independent risk factors for the prognosis of gastric cancer (Table 3). The overall survival of the entire cohort was shown in Figure 1. The results showed that duration of fever less than 48 h did not influence the survival of gastric cancer patients (*P* = 0.283). However, duration of fever over 48h was significantly associated with decreased prognosis of gastric cancer patients (*P* < 0.001).

**Table 2:** Univariate analysis of risk factors for the prognosis of entire cohort.

Prognostic factors	β	Hazard ratio (95% CI)	*P* value
Gender	0.078	1.081(0.916-1.276)	0.355
Age	0.312	1.366(1.190-1.569)	<0.001
Tumor location	-0.043	0.958 (0.893-1.028)	0.232
Tumor size	1.123	3.073(2.673-3.532)	<0.001
Type of resection	-0.554	0.575(0.511-0.647)	<0.001
Pathological type	0.457	1.580(1.434-1.741)	<0.001
Tumor depth	0.800	2.225(2.041-2.426)	<0.001
Lymph node metastasis	0.719	2.052(1.924-2.190)	<0.001
Tumor stage	1.274	3.573(3.156-4.046)	<0.001
Fever	0.172	1.187(1.079-1.307)	<0.001

**Table 3:** Multivariate analysis of risk factors for the prognosis of entire cohort.

Prognostic factors	β	Hazard ratio (95% CI)	*P* value
Age	0.333	1.395 (1.214-1.602)	<0.001
Tumor size	0.425	1.529 (1.318-1.775)	<0.001
Tumor depth	0.448	1.565 (1.416-1.730)	<0.001
Lymph node metastasis	0.520	1.681 (1.563-1.809)	<0.001
Fever	0.206	1.229 (1.116-1.353)	<0.001

**Figure 2:**
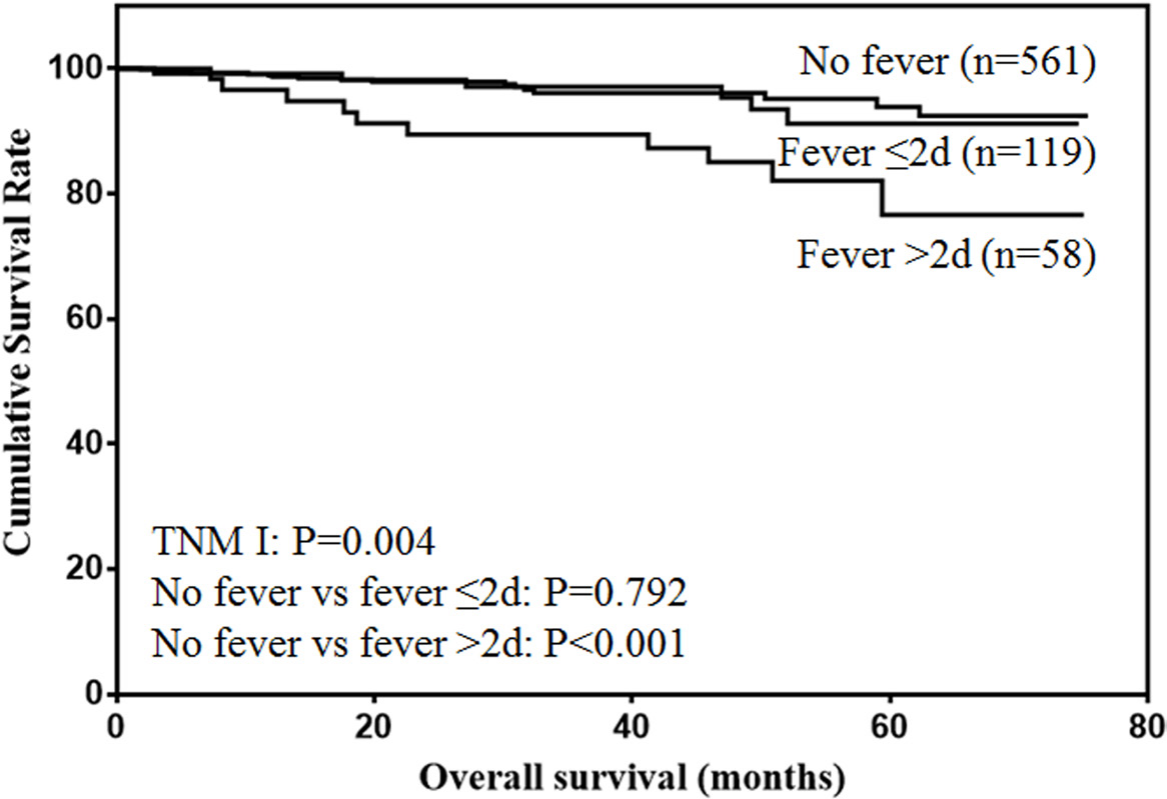
Overall survival of stage I gastric cancer patients

**Figure 3:**
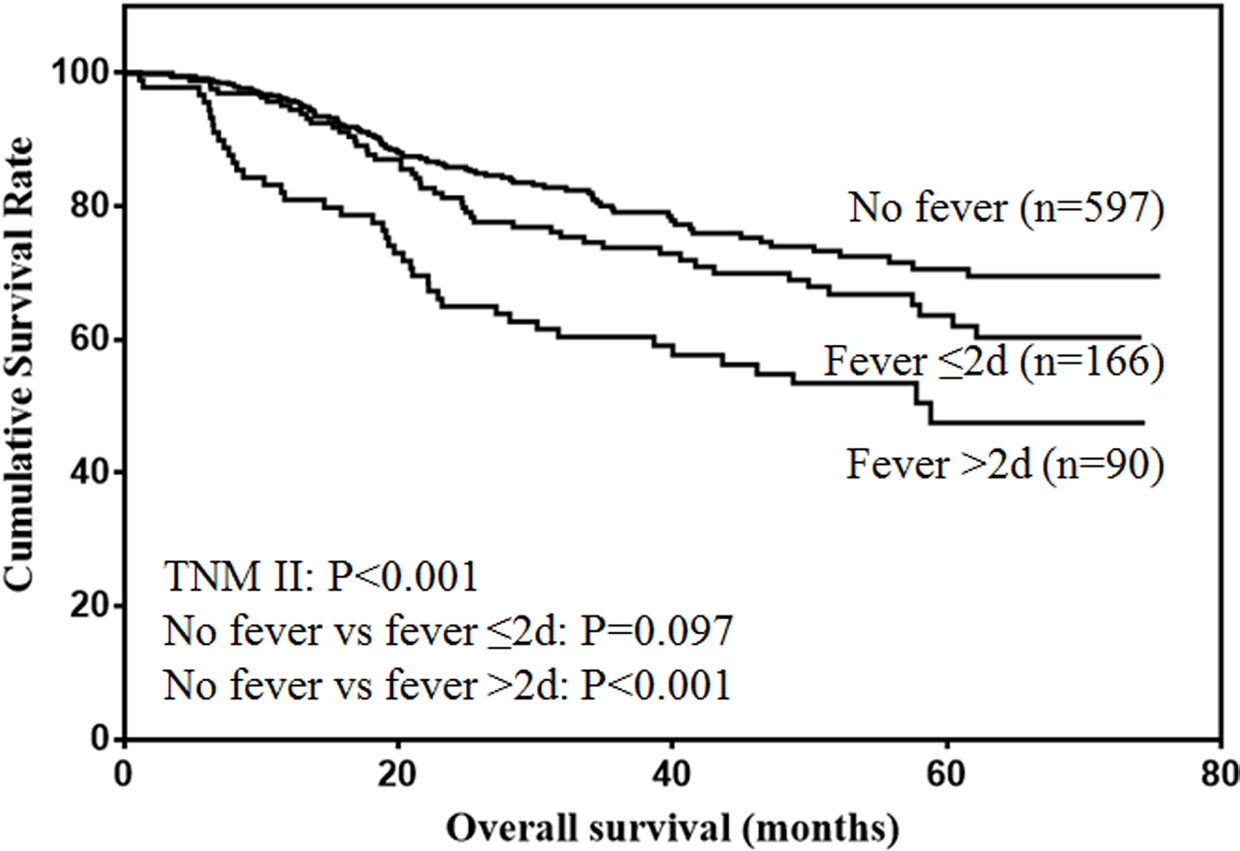
Overall survival of stage II gastric cancer patients

**Figure 4:**
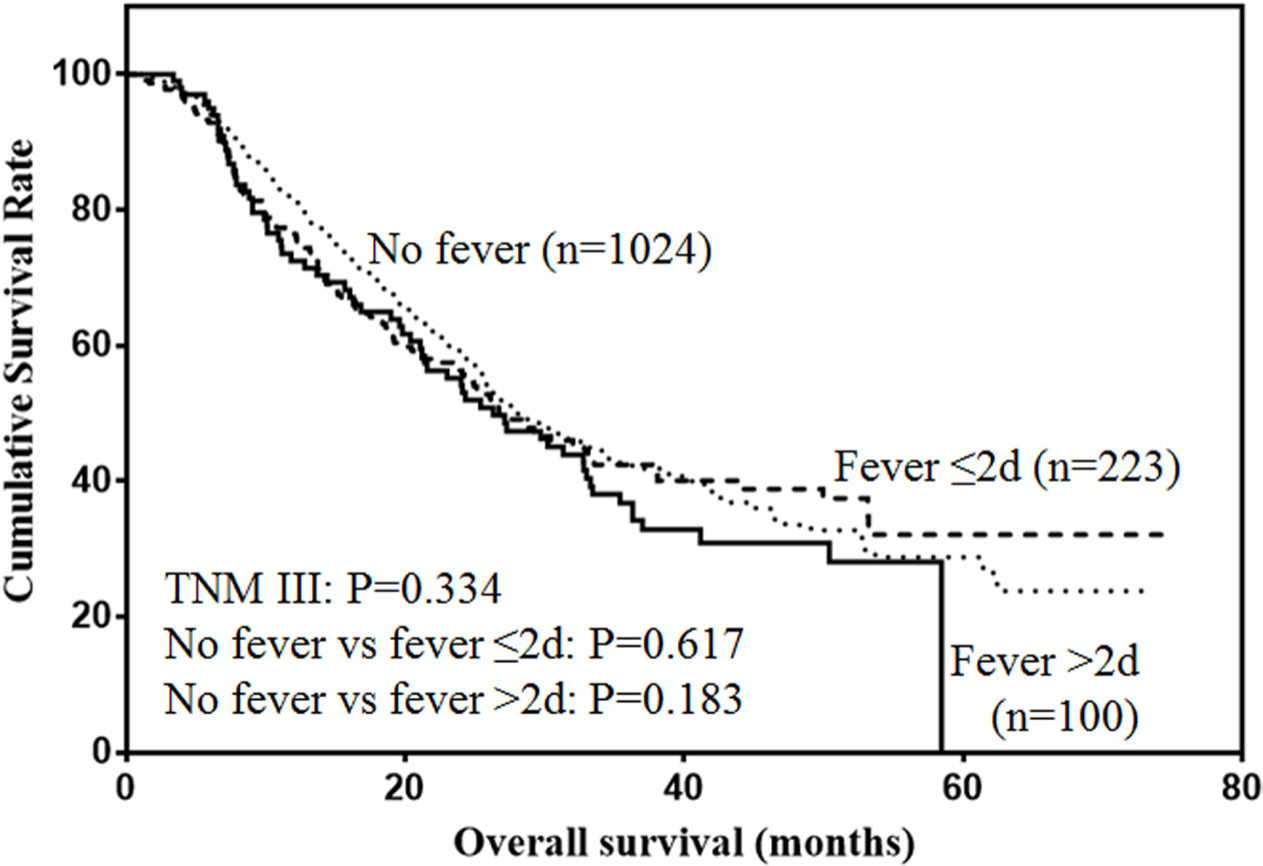
Overall survival of stage III gastric cancer patients

The prognostic value of postoperative fever according to different tumor stages were analyzed. For stage I patients (Figure 2), only duration of fever over 48h was significantly associated with decreased survival (*P* < 0.001). For stage II patients (Figure 3), although duration of fever less than 48 h was associated with decreased survival, the difference was not significant (*P* = 0.097). Duration of fever over 48h was significantly associated with decreased survival of gastric cancer patients (*P* < 0.001). For stage III patients (Figure 4), postoperative fever was not associated with the prognosis of gastric cancer (*P* = 0.334).

**Figure 5:**
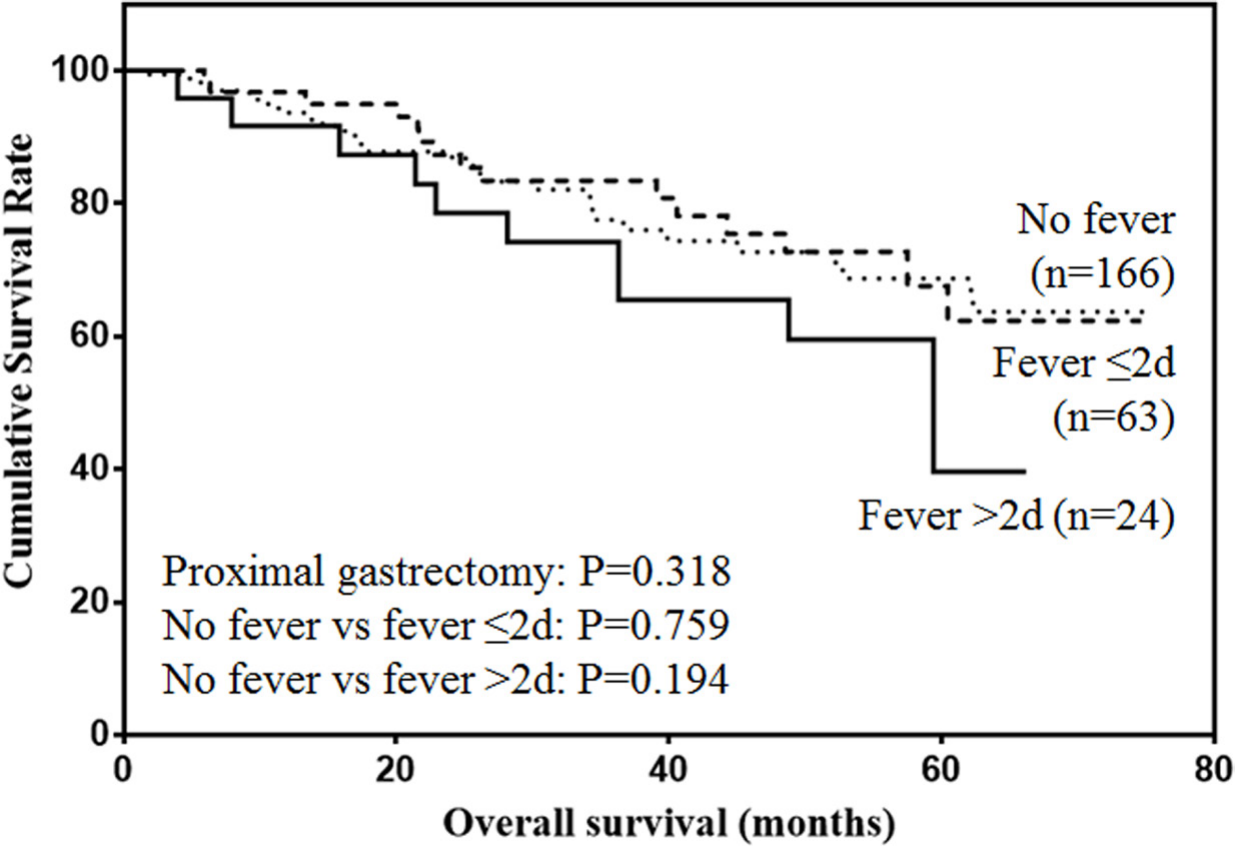
Overall survival of patients with proximal gastrectomy

**Figure 6:**
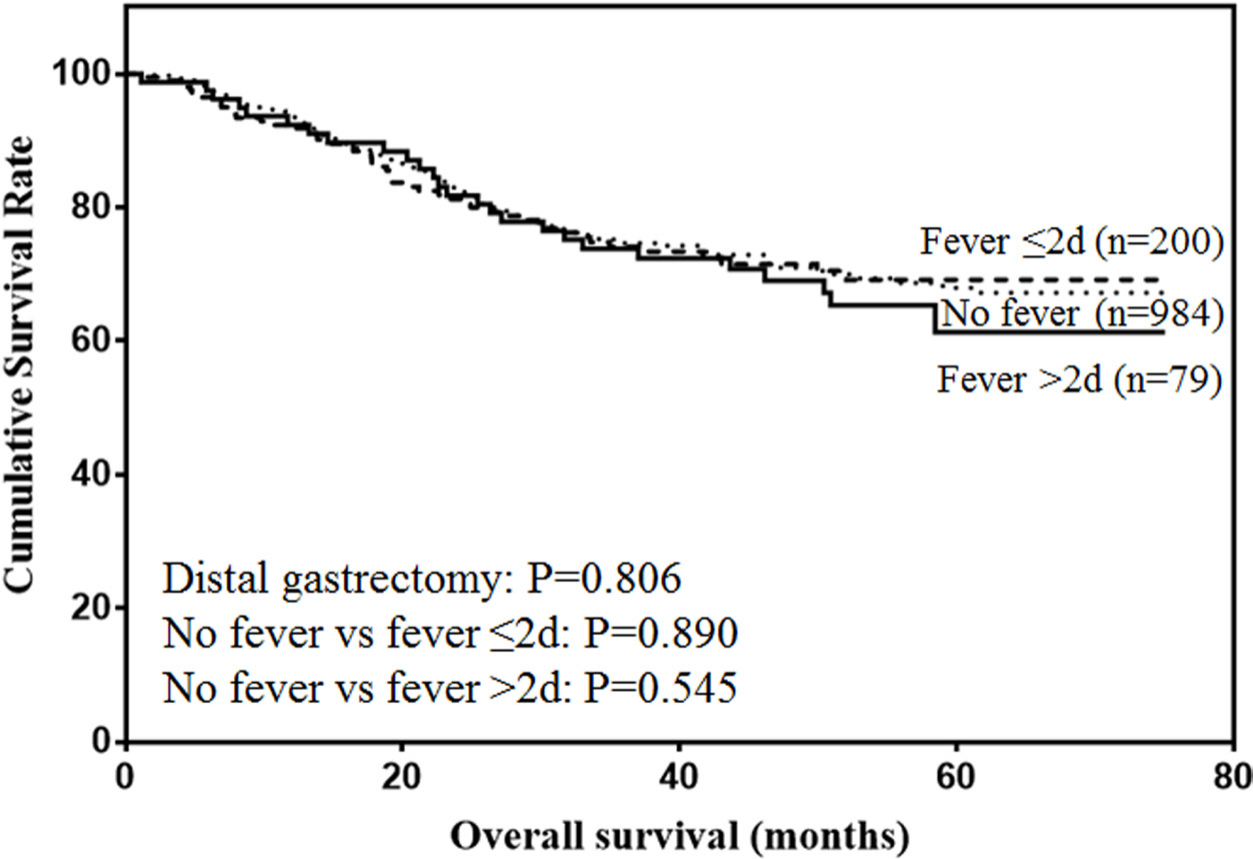
Overall survival of patients with distal gastrectomy

**Figure 7:**
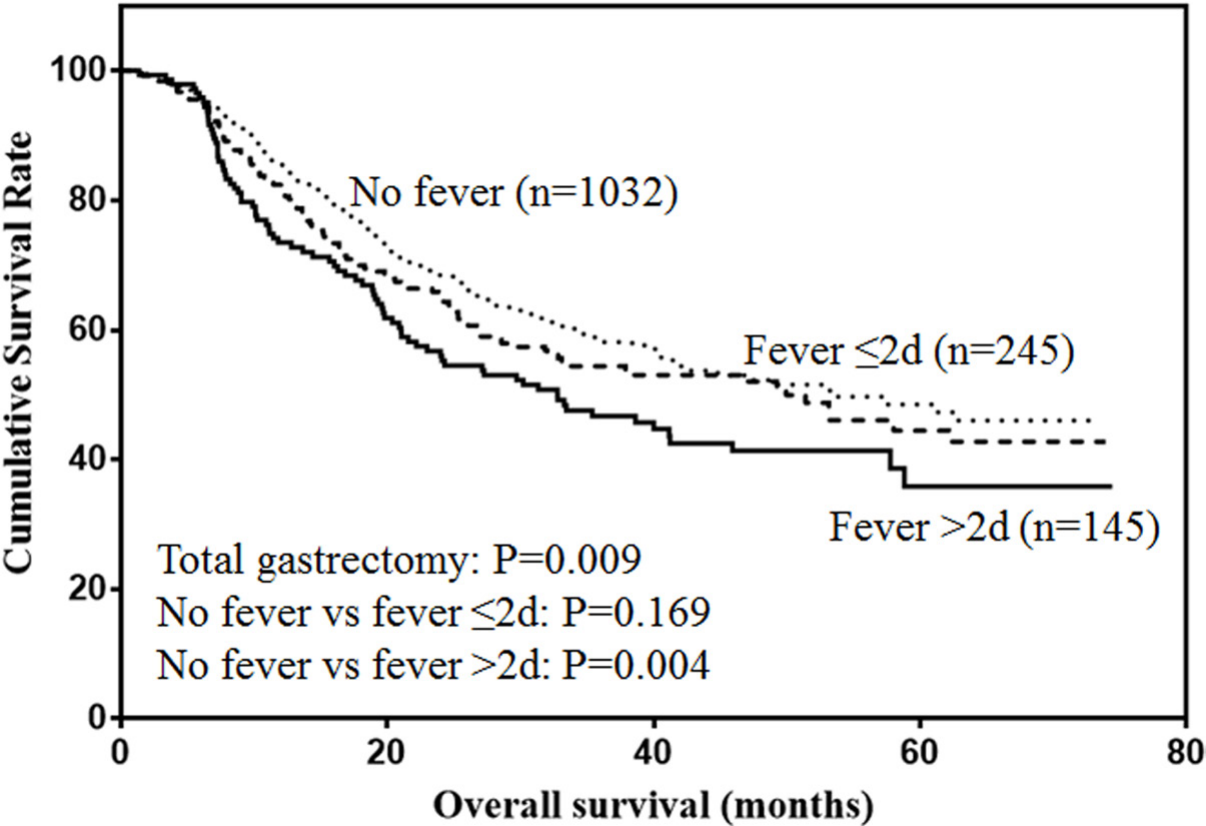
Overall survival of patients with total gastrectomy

The prognostic value of postoperative fever according to the type of gastrectomy were also analyzed. For proximal gastrectomy (Figure 5), patients with postoperative fever lasting more than 48h was associated with lower prognosis, although the difference was not significant (*P* = 0.138). For distal gastrectomy (Figure 6), postoperative fever was not associated with the prognosis of gastric cancer (*P* = 0.806). For total gastrectomy (Figure 7), only duration of fever over 48h was significantly associated with decreased survival (*P* = 0.004).

## CONCLUSIONS

Postoperative fever is one of the host responses to surgery. Thus, postoperative fever is very common in clinic. However, the association between postoperative fever and survival of gastric cancer patients is unclear. The present study found that postoperative fever was an independent prognostic predictor for gastric cancer patients.

The prognostic value of postoperative fever has only been reported in a few studies including breast cancer and colorectal cancer. Teucher et al. reported that fever could significantly increase the relative risk recurrence in stage II/III breast cancer [6]. Yan et al. also reported that fever may contribute to recurrence in node negative breast cancer patients, and postoperative fever was an independent risk factor for relapse free survival [5]. However, the findings in colorectal cancer were controversial. In 1983, Nowacki et al. for the first time reported that postoperative fever lasting two or more days was the most unfavorable prognostic factor for colorectal cancer patients [9]. However, Fucini et al. demonstrated that postoperative fever did not significantly influence the survival of colorectal cancer patients [8]. The latest report containing 2311 colorectal cancer patients also demonstrated that postoperative fever was not an independent risk factor for disease specific and overall survival [7]. Up to date, no study has reported the association between the postoperative fever and prognosis of gastric cancer patients. For the first time, our present study containing 2938 cases found that postoperative fever was an independent prognostic predictor for gastric cancer, especially for stage I and II patients. Although postoperative fever was associated with the decreased survival of gastric cancer patients, the prognostic value was limited to some extent. As the prognosis of stage III gastric cancer was extremely poor, postoperative fever may have lost its prognostic value for stage III tumors.

In the previous reports, postoperative fever was defined variously, such as 1) temperature >100.0°F (37.8°C), lasting two or more days and beginning on the fourth postoperative day [8, 9], 2) temperature > 37.5°C from the second postoperative day [6], 3) temperature ≥101.0°F (38.3°C) during the postoperative period [7], 4) oral temperature ≥100.4°F (38°C) in one week after surgery [5]. The current study defined postoperative fever as one measurement of axillary temperature ≥100.4°F (38°C) from the first postoperative day. The different definition of postoperative fever may finally result in different incidence and prognostic significance of fever in cancer patients.

The causes of fever may attribute to infectious or noninfectious events. The most common etiology of postoperative fever is associated with the normal thermoregulatory response without infection in vivo [10]. It reflects the host responses to surgery. Unexplained postoperative fever is characterized by early onset of lower maximum temperature with duration of less than three days [11, 12]. On the other hand, continuous postoperative fever over 72 h or temperature over 38.8°C strongly indicated the occurrence of new complications [7, 13]. In our present study, infectious and noninfectious postoperative fever were both included in the analysis, and the prognostic value of infectious and noninfectious fever were not analyzed separately. This was a limitation in our present study.

The mechanisms of the influence of postoperative fever on survival of cancer patients is still poorly understood. Serum IL-6 is a thermoregulatory factor during surgery and general anesthesia [14], and is a major endogenous mediator of fever [15]. Levels of serum IL-6 is also elevated in a variety of tumors [16-18]. Thus, postoperative fever may be partly due to the elevated serum IL-6 level in vivo [19]. Experimental studies demonstrated that IL-6 could promote proliferation, invasion and metastasis of tumor cells [20, 21]. Thus, the elevated serum IL-6 level in cancer patients after surgery may promote the recurrence of patients with postoperative fever. Moreover, serum IL-6 produced by tumor cells could increase the drug resistance to chemotherapy [22], which may also contribute to tumor recurrence after surgery. Unfortunately, data about the recurrence of gastric cancer patients was lacking in our present study.

There were some limitations in our present study. Firstly, it was a retrospective analysis with single center’s experience. Multi-center studies are needed to verify the predictive value of postoperative fever in gastric cancer patients. Secondly, all cases of fever were included in the analysis, the prognostic value of infectious and noninfectious fever were not analyzed separately. Because infectious and noninfectious fever may have different prognostic value for gastric cancer patients. Thirdly, fever was not classified into different degrees according to the maximum temperature. Fourth, the influence of physical or medical treatments to reduce body temperature on the survival of patients were not analyzed.

In conclusion, duration of postoperative fever over 48 h was an independent risk factor for the prognosis of gastric cancer patients, especially for stage I/II gastric cancer and patients with total gastrectomy.

## MATERIALS AND METHODS

This study was performed in the Xijing Hospital of Digestive Diseases affiliated to the Fourth Military Medical University. From September 2008 to March 2015, a total of 2938 gastric cancer patients in our department were enrolled in the present study. The inclusion criteria were listed as follows: 1. without other malignant tumor, 2. without distant metastasis, 3. without neoadjuvant chemotherapy, 4. with radical D2 gastrectomy, 5. with temperature records, 6. with follow up data. This study was approved by the Ethics Committee of Xijing Hospital, and written informed consent was obtained from all patients before surgery.

All patients were treated with proximal, distal or total gastrectomy with D2 lymphadenectomy. The surgical procedure was based on the recommendations of the Japanese Gastric Cancer Treatment Guidelines [10]. The depth of primary tumor and degree of lymph node involvement were defined according to the TNM classification.

The body temperature was measured with an axillary thermometer four times a day after surgery. Fever was defined as axillary temperature ≥ 38.0 degrees centigrade. Duration of fever were also recorded. Clinicopathological data including gender, age, tumor location, tumor size, type of resection, Borrmann type, pathological type, tumor depth, lymph node metastasis and tumor stage were collected. The patients were followed up till November 2015 every 3 months.

Data were processed using SPSS 22.0 for Windows (SPSS Inc., Chicago, IL, USA). Discrete variables were analyzed using Chi-square test or Fisher’s exact test. Significant risk factors identified by univariate analysis were further assessed by multivariate analysis using the Cox’s proportional hazards regression model. Overall survival was analyzed by Kaplan-Meier method. The P value was considered to be statistically significant at 5% level.
